# Synthesis and crystal structure determination of aluminium hydroxide methane­sulfonate, Al(OH)(CH_3_SO_3_)_2_

**DOI:** 10.1107/S2056989026005578

**Published:** 2026-06-05

**Authors:** Eric Gabilondo, P. Shiv Halasyamani

**Affiliations:** a School of Chemistry, University College Dublin, Belfield, Dublin 4, Ireland; b Department of Chemistry University of Houston, 3585 Cullen Blvd Room 112 Houston TX 77204-5003 USA; Tokyo University of Science, Japan

**Keywords:** crystal structure, methane­sulfonic acid, aluminium, hydroxide, crystal growth

## Abstract

*catena*-Poly[aluminium(III)-μ-hydroxido-κ^2^*O*:*O*-di-μ-methane­sulfonato-κ^4^*O*:*O*′], [Al(OH)(CH_3_SO_3_)_2_]_*n*_, was prepared by mild hydro­thermal synthesis at 523 K and crystallizes as millimetre-sized clear and colourless needles. The asymmetric unit contains half of the repeating structure, one-dimensional (AlO_6_)_∞_ chains corner sharing through the axial hydroxyl group with a bend of ∼137^ο^. The extended structure shows the chains are connected through weak meth­yl–hydrogen bonding in the [001] direction and strong hydrox­yl–hydrogen bonding in the [100] direction. This is the second aluminium-based methane­sulfonate salt reported to date.

## Abstract 2

An aluminium hydroxide methane­sulfonate salt, Al(OH)(CH_3_SO_3_)_2_, crystallizes with one-dimensional chains of AlO_6_ connected via hydrogen bonding.

## 1. Chemical context

Crystals containing the methane­sulfonate anion, CH_3_SO_3_^−^, have attracted inter­est as potential linear and non-linear optical crystals (Tian *et al.*, 2023 ▸; Gabilondo & Halasyamani, 2025 ▸). However, there are relatively few crystal structures reported compared to other anionic groups such as Cl^−^, F^−^, SO_4_^2−^, or PO_4_^3−^, to name a few, rendering structure prediction a challenge. Herein, we report an aluminium-based methane­sulfonate salt, Al(OH)(CH_3_SO_3_)_2_ (**I**). The title compound is the second Al-based methane­sulfonate to be discovered alongside Al(CH_3_SO_3_)_3_(H_2_O)_6_, (**II;** Trella & Frank, 2012 ▸).
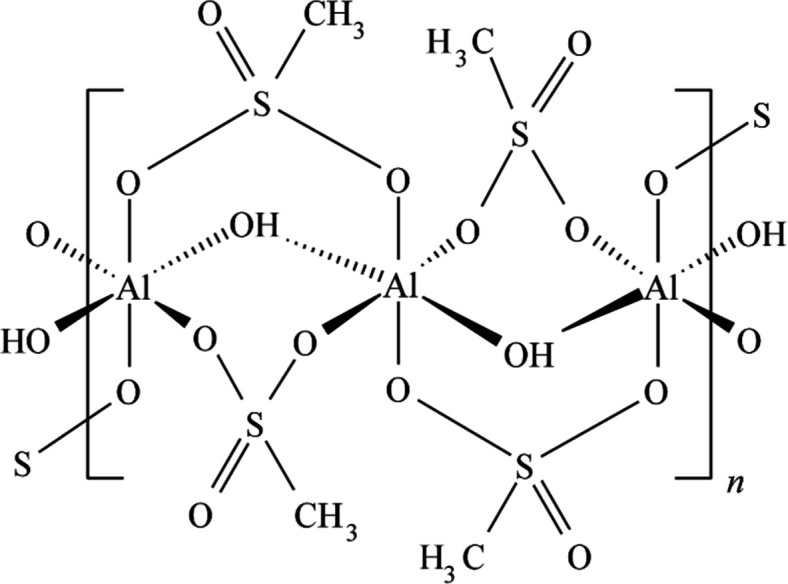


## 2. Structural commentary

**Figure 1:**
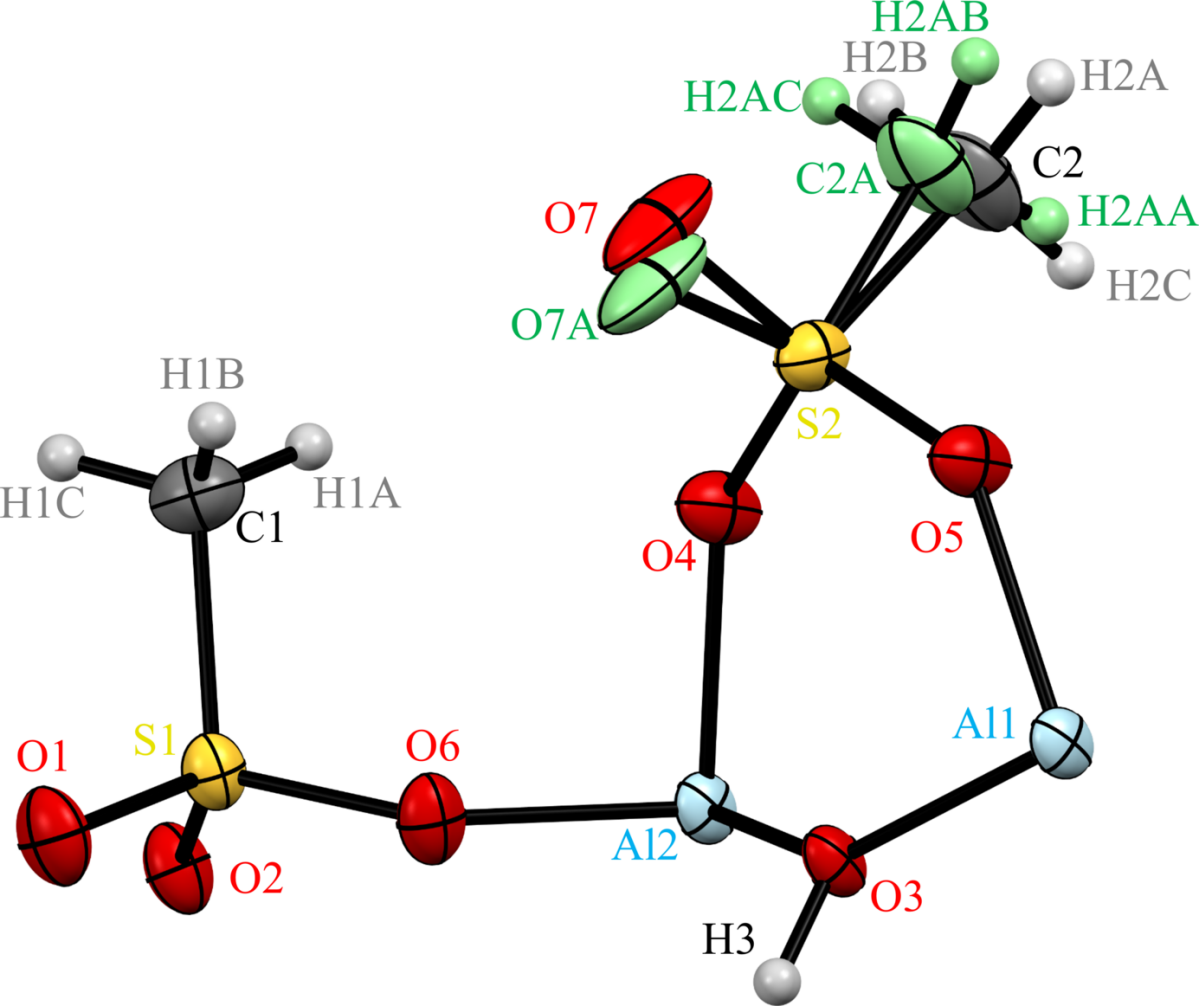
The asymmetric unit of **I** with displacement ellipsoids shown at the 50% probability level. The terminal oxygen (O7) and methyl (C2, H2*A*, H2*B*, H2*C*) groups are disordered with refined occupancies of 0.68 (3)/0.32 (3). The minor residue is shown in green.

Compound **I** crystallizes in the triclinic space group *P*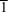. The asymmetric unit, shown in Fig. 1 ▸, contains half of the two repeating AlO_6_ octa­hedra, which are corner sharing at the axial positions through a hydroxyl bridge (O3—H3). Each octa­hedron is additionally bridged at the equatorial positions by two methane­sulfonate anion groups (O6/S1/O2 and O5/S2/O4). The two Al atoms differ by Wyckoff position 1*f* and 1*h*, for Al1 and Al2, respectively. The AlO_6_ octa­hedra have a small axial compression (∼4%) with an average bond length of (axial) 1.8394 (14) Å and (equatorial) 1.9165 (15) Å. There is little angular distortion from the ideal values of 90 (1) and 180°. The methane­sulfonate groups are largely undistorted and have similar geometries to those in previous reports (Wei & Hingerty, 1981 ▸). For example, the average S—O single bond length is 1.473 (1) Å, S=O is 1.439 (3) Å, and S—C is 1.734 (2) Å, compared to literature values of 1.461 (1), 1.452 (1) and 1.754 (2) Å, respectively. One methane­sulfonate group (S2) is disordered with refined occupancies of 0.68 (3)/0.32 (3). There is a minor rotation of the O7—S2—C2 angle of 11.7 (12)° between the two residues with S2 unaffected. The cause is likely weak inter­layer hydrogen bonding, discussed below and not uncommon in metal methane­sulfonates (Singh *et al.*, 2020 ▸; Wickleder & Müller, 2004 ▸). The structure of **I** is in contrast to the known **II** that has isolated Al(OH_2_)_6_ octa­hedra bridged via hydrogen bonding to CH_3_SO_3_^−^ anions, with no direct coordination of the octa­hedra nor the CH_3_SO_3_^−^ groups. The bond-valence sum for each atom (Brown 2009 ▸) is consistent with the expected oxidation states of Al^III^, S^VI^, O^2−^, and C^IV^, with average experimental values of 3.155 (5), 5.83 (4), 2.0 (1), and 4.136 (3), respectively, and supports reasonable hydrogen-atom assignments.

## 3. Supra­molecular features

**Figure 2:**
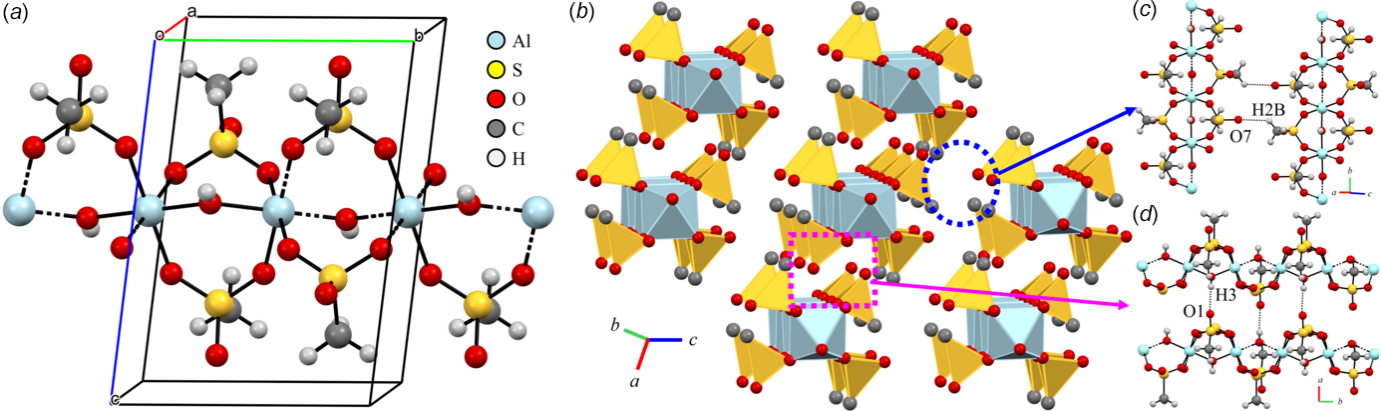
(*a*) Packing diagram and unit cell for **I** and (*b*) polyhedral extended structure of one-dimensional chains. AlO_6_ chains are connected via (*c*) weak hydrogen bonding (C2—H2*B*⋯O7) bonding in the [001] direction and (*d*) with traditional hydrogen bonding (O3—H3⋯O1) in the [100] direction. Disorder is removed for clarity.

The unit cell and packing diagram of **I** is shown in Fig. 2 ▸*a* with primary supra­molecular structural motifs in Fig. 2 ▸*b*–*d*. As shown in Fig. 2 ▸*a*, the unit cell contains two asymmetric units to complete a one-dimensional chain of AlO_6_ octa­hedra. The chains are corner-sharing through the OH group at the axial positions and rotate by an Al—O—Al angle of 134.57 (7)°. Fig. 2 ▸*b* highlights the extended structure as isolated one-dimensional chains connected *via* hydrogen bonding (Table 1 ▸). The chains are connected weakly in the [001] direction through hydrogen bonding (Fig. 2 ▸*c*) between the terminal S2=O7 group and the methyl group on C2. The weak hydrogen bonding is likely responsible for the disorder of C2, S2, and O7, with weak inter­actions not ‘locking’ the terminal groups in place. Meanwhile, stronger and more traditional hydrogen bonding connects the chains in the [100] direction through the hydroxyl group from O3—H3⋯O1 (Fig. 2 ▸*d*). The relative strength of the inter­chain hydrogen bonding is exemplified by the Al⋯Al distances of 9.7677 (15) Å in the [001] direction and 6.5099 (11) Å in the [100] direction, corresponding to the unit-cell parameters *c* and *a*, respectively. The extended structure of **I** again contrasts with the structure of **II** by having direct connectivity through one-dimensional chains of AlO_6_ octa­hedra rather than the 0D structure of **II** held together by a hydrogen-bonding network.

## 4. Database survey

The Cambridge Structural Database (CSD, accessed May 2026; Groom *et al.*, 2016 ▸) contains 54 metal–methane­sulfonates and the Inorganic Crystalline Structure Database (ICSD; Zagorac *et al.*, 2019 ▸) contains 21. Only one of these contains Al, as highlighted earlier: **II** Al(CH_3_SO_3_)_3_(H_2_O)_6_ (LEHREX; Trella & Frank, 2012 ▸). Compound **I** is the first reported hydroxide-bridged aluminium methane­sulfonate containing one-dimensional, corner-sharing AlO_6_ chains. Compound **I** is not isostructural with other reported metal–methane­sulfonate hydroxides, even the empirically similar Sc(OH)(CH_3_SO_3_)_2_ (ESARAR; Wickleder & Müller, 2004 ▸).

## 5. Synthesis and crystallization

Compound **I** was synthesized by a hydro­thermal route. 1.322 g (3.5 mmol) of aluminium nitrate nona­hydrate [Al(NO_3_)_3_(H_2_O)_9_, Alfa Aesar, 98%] were added to 1 mL of methane­sulfonic acid (4.9 mmol, 70 *w*/*w*% in H_2_O, Thermo Fisher) inside a Teflon-lined autoclave prior to sealing and placing in a muffle furnace. The furnace was heated to 523 K at a rate of 1 K min^−1^, held for 72 h, then radiatively cooled to room temperature. Solid products were collected via vacuum filtration followed by rinsing with aceto­nitrile to remove solvent. Millimetre-sized, clear and colourless needle-shaped crystals were separated mechanically from the other insoluble and amorphous solid products. Crystals were dried overnight in a vacuum desiccator prior to further analysis. Yield was ∼56% with respect to Al. The crystals are slightly hygroscopic and slowly decompose to **II** in air over the course of several weeks or rapidly upon grinding.

## 6. Refinement

Crystal data, data collection and structure refinement details are summarized in Table 2 ▸. Methyl-H atoms were refined using a riding model with ideal tetra­hedral angles once identified using difference maps. The hydroxyl-H atom (H3) was similarly identified by both difference maps and a low bond-valence sum around O3 of 1.2 v.u. (Brown 2009 ▸). The O3—H3 bond was restrained to 0.97 (1) Å [*U*_iso_(H) = 1.5*U*_eq_(O)] and restrained to be equidistant from Al1 and Al2 to maintain the ideal OH geometry. Restraints were adopted because the O3—H3 bond became unusually short unrestrained (∼0.73 Å). The terminal methane­sulfonate unit was treated as a disordered residue due to unusually large anisotropic displacement parameters on C2 and O7, with refined occupancies of 0.68 (3)/0.32 (3).

## Supplementary Material

Crystal structure: contains datablock(s) I. DOI: 10.1107/S2056989026005578/jp2028sup1.cif

Structure factors: contains datablock(s) I. DOI: 10.1107/S2056989026005578/jp2028Isup2.hkl

CCDC reference: 2541524

Additional supporting information:  crystallographic information; 3D view; checkCIF report

## Figures and Tables

**Table 1:** Hydrogen-bond geometry (Å, °) Hydrogen-bonding distances refer to the major residue.

*D*—H⋯*A*	*D*—H	H⋯*A*	*D*⋯*A*	*D*—H⋯*A*
C1—H1*B*⋯O7^i^	0.96	2.62	3.304 (15)	129
C2—H2*B*⋯O7	0.96	2.72	3.641 (16)	162
O3—H3⋯O1^ii^	0.94 (1)	1.87 (1)	2.802 (3)	171 (2)

Symmetry codes: (i) 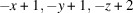; (ii) 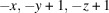.

**Table 2:** Experimental details

Crystal data	
Chemical formula	[Al(OH)(CH_3_SO_3_)_2_]
*M*_r_	234.18
Crystal system, space group	Triclinic, *P* 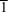
Temperature (K)	301
*a*, *b*, *c* (Å)	6.5099 (10), 6.7869 (10), 9.7677 (15)
α, β, γ (°)	94.712 (6), 109.253 (6), 90.177 (6)
*V* (Å^3^)	405.83 (11)
*Z*	2
Radiation type	Mo *K*α
μ (mm^−1^)	0.76
Crystal size (mm)	0.25 × 0.03 × 0.03

Data collection	
Diffractometer	Bruker APEXII CCD
Absorption correction	Multi-scan (*SADABS*; Krause *et al.*, 2015 ▸)
*T*_min_, *T*_max_	0.674, 0.746
No. of measured, independent and observed [*I* > 2σ(*I*)] reflections	15679, 2007, 1707
*R*_int_	0.050
(sin θ/λ)_max_ (Å^−1^)	0.667

Refinement	
*R*[*F*^2^ > 2σ(*F*^2^)], *wR*(*F*^2^), *S*	0.034, 0.087, 1.07
No. of reflections	2007
No. of parameters	125
No. of restraints	2
H-atom treatment	H atoms treated by a mixture of independent and constrained refinement
Δρ_max_, Δρ_min_ (e Å^−3^)	0.43, −0.41

Computer programs: *APEX5* (Bruker, 2023 ▸), *SAINT* (Bruker, 2008 ▸), *SHELXT2018/2* (Sheldrick, 2015*a* ▸), *SHELXL2019/2* (Sheldrick, 2015*b* ▸) and *Mercury* (Macrae *et al.*, 2020 ▸).

## supplementary crystallographic information

### catena-Poly[aluminium(III)-µ-hydroxido-κ2O:O-di-µ-methanesulfonato-κ4O:O'] Crystal data

**Table:**

[Al(OH)(CH_3_SO_3_)_2_]	*Z* = 2
*M**_r_* = 234.18	*F*(000) = 240
Triclinic, *P*1	*D*_x_ = 1.916 Mg m^−^^3^
*a* = 6.5099 (10) Å	Mo *K*α radiation, λ = 0.71073 Å
*b* = 6.7869 (10) Å	Cell parameters from 4784 reflections
*c* = 9.7677 (15) Å	θ = 2.2–27.8°
α = 94.712 (6)°	µ = 0.76 mm^−^^1^
β = 109.253 (6)°	*T* = 301 K
γ = 90.177 (6)°	Needle, colourless
*V* = 405.83 (11) Å^3^	0.25 × 0.03 × 0.03 mm

### catena-Poly[aluminium(III)-µ-hydroxido-κ2O:O-di-µ-methanesulfonato-κ4O:O'] Data collection

**Table:**

Bruker APEXII CCD diffractometer	1707 reflections with *I* > 2σ(*I*)
φ and ω scans	*R*_int_ = 0.050
Absorption correction: multi-scan (SADABS; Krause *et al.*, 2015)	θ_max_ = 28.3°, θ_min_ = 2.2°
*T*_min_ = 0.674, *T*_max_ = 0.746	*h* = −8→8
15679 measured reflections	*k* = −9→9
2007 independent reflections	*l* = −12→13

### catena-Poly[aluminium(III)-µ-hydroxido-κ2O:O-di-µ-methanesulfonato-κ4O:O'] Refinement

**Table:**

Refinement on *F*^2^	Primary atom site location: dual
Least-squares matrix: full	Hydrogen site location: mixed
*R*[*F*^2^ > 2σ(*F*^2^)] = 0.034	H atoms treated by a mixture of independent and constrained refinement
*wR*(*F*^2^) = 0.087	*w* = 1/[σ^2^(*F*_o_^2^) + (0.0417*P*)^2^ + 0.2379*P*] where *P* = (*F*_o_^2^ + 2*F*_c_^2^)/3
*S* = 1.07	(Δ/σ)_max_ < 0.001
2007 reflections	Δρ_max_ = 0.43 e Å^−^^3^
125 parameters	Δρ_min_ = −0.41 e Å^−^^3^
2 restraints	

### catena-Poly[aluminium(III)-µ-hydroxido-κ2O:O-di-µ-methanesulfonato-κ4O:O'] Special details

**Table:**

Geometry. All esds (except the esd in the dihedral angle between two l.s. planes) are estimated using the full covariance matrix. The cell esds are taken into account individually in the estimation of esds in distances, angles and torsion angles; correlations between esds in cell parameters are only used when they are defined by crystal symmetry. An approximate (isotropic) treatment of cell esds is used for estimating esds involving l.s. planes.

### catena-Poly[aluminium(III)-µ-hydroxido-κ2O:O-di-µ-methanesulfonato-κ4O:O'] Fractional atomic coordinates and isotropic or equivalent isotropic displacement parameters (Å^2^)

**Table:**

	*x*	*y*	*z*	*U*_iso_*/*U*_eq_	Occ. (<1)
S1	0.26117 (8)	0.76941 (7)	0.66932 (5)	0.01853 (14)	
S2	0.77597 (9)	0.28304 (7)	0.77275 (5)	0.02129 (15)	
Al1	0.500000	0.000000	0.500000	0.0166 (2)	
Al2	0.500000	0.500000	0.500000	0.0165 (2)	
O1	0.0586 (3)	0.7731 (2)	0.69736 (18)	0.0284 (4)	
O2	0.2868 (3)	0.9380 (2)	0.58889 (17)	0.0235 (3)	
O3	0.3839 (2)	0.24615 (19)	0.47067 (15)	0.0171 (3)	
H3	0.2321 (17)	0.2411 (18)	0.424 (2)	0.026*	
O4	0.7021 (3)	0.4503 (2)	0.68421 (16)	0.0247 (4)	
O5	0.6994 (3)	0.0937 (2)	0.68568 (16)	0.0242 (3)	
O6	0.2883 (2)	0.5812 (2)	0.59119 (16)	0.0229 (3)	
C1	0.4736 (4)	0.7916 (4)	0.8366 (2)	0.0319 (5)	
H1A	0.610534	0.780972	0.819857	0.048*	
H1B	0.457894	0.688125	0.894139	0.048*	
H1C	0.468552	0.917766	0.887359	0.048*	
O7	0.740 (3)	0.3028 (15)	0.9115 (14)	0.0442 (18)	0.68 (3)
C2	1.070 (2)	0.2877 (18)	0.8079 (14)	0.0376 (19)	0.68 (3)
H2A	1.131846	0.175313	0.858649	0.056*	0.68 (3)
H2B	1.134113	0.406980	0.866437	0.056*	0.68 (3)
H2C	1.097876	0.283080	0.717226	0.056*	0.68 (3)
C2A	1.035 (4)	0.287 (4)	0.837 (3)	0.0376 (19)	0.32 (3)
H2AA	1.092830	0.265748	0.758451	0.056*	0.32 (3)
H2AB	1.081138	0.184014	0.901309	0.056*	0.32 (3)
H2AC	1.087015	0.412787	0.889476	0.056*	0.32 (3)
O7A	0.683 (4)	0.294 (4)	0.881 (2)	0.0442 (18)	0.32 (3)

### catena-Poly[aluminium(III)-µ-hydroxido-κ2O:O-di-µ-methanesulfonato-κ4O:O'] Atomic displacement parameters (Å^2^)

**Table:**

	*U*^11^	*U*^22^	*U*^33^	*U*^12^	*U*^13^	*U*^23^
S1	0.0180 (3)	0.0142 (2)	0.0248 (3)	−0.00089 (18)	0.00896 (19)	0.00137 (19)
S2	0.0278 (3)	0.0128 (2)	0.0201 (3)	−0.0013 (2)	0.0036 (2)	0.00119 (18)
Al1	0.0182 (4)	0.0076 (4)	0.0228 (4)	−0.0012 (3)	0.0052 (3)	0.0016 (3)
Al2	0.0181 (4)	0.0077 (4)	0.0228 (4)	−0.0012 (3)	0.0057 (3)	0.0011 (3)
O1	0.0230 (8)	0.0260 (8)	0.0422 (9)	0.0011 (6)	0.0183 (7)	0.0042 (7)
O2	0.0246 (8)	0.0143 (7)	0.0356 (8)	0.0023 (6)	0.0145 (7)	0.0064 (6)
O3	0.0154 (7)	0.0090 (6)	0.0255 (7)	0.0000 (5)	0.0044 (6)	0.0021 (5)
O4	0.0286 (8)	0.0114 (7)	0.0276 (8)	−0.0017 (6)	0.0002 (6)	0.0026 (6)
O5	0.0277 (8)	0.0116 (7)	0.0266 (7)	−0.0010 (6)	−0.0002 (6)	0.0015 (6)
O6	0.0239 (8)	0.0132 (7)	0.0337 (8)	−0.0031 (6)	0.0132 (6)	−0.0008 (6)
C1	0.0322 (13)	0.0359 (13)	0.0244 (11)	−0.0011 (10)	0.0053 (9)	0.0013 (9)
O7	0.078 (6)	0.0363 (14)	0.018 (4)	−0.010 (3)	0.017 (3)	−0.003 (2)
C2	0.017 (4)	0.0316 (15)	0.060 (5)	−0.003 (2)	0.007 (2)	0.002 (3)
C2A	0.017 (4)	0.0316 (15)	0.060 (5)	−0.003 (2)	0.007 (2)	0.002 (3)
O7A	0.078 (6)	0.0363 (14)	0.018 (4)	−0.010 (3)	0.017 (3)	−0.003 (2)

### catena-Poly[aluminium(III)-µ-hydroxido-κ2O:O-di-µ-methanesulfonato-κ4O:O'] Geometric parameters (Å, º)

**Table:**

S1—O1	1.4324 (16)	Al2—O3	1.8411 (13)
S1—O6	1.4749 (15)	Al2—O3^ii^	1.8411 (13)
S1—O2	1.4790 (15)	Al2—O4^ii^	1.9037 (14)
S1—C1	1.753 (2)	Al2—O4	1.9037 (14)
S2—O7A	1.38 (3)	Al2—O6	1.9315 (15)
S2—O7	1.444 (10)	Al2—O6^ii^	1.9315 (15)
S2—O4	1.4691 (15)	O3—H3	0.941 (10)
S2—O5	1.4696 (15)	C1—H1A	0.9600
S2—C2A	1.59 (2)	C1—H1B	0.9600
S2—C2	1.828 (14)	C1—H1C	0.9600
Al1—O3	1.8407 (13)	C2—H2A	0.9600
Al1—O3^i^	1.8407 (13)	C2—H2B	0.9600
Al1—O5	1.9056 (14)	C2—H2C	0.9600
Al1—O5^i^	1.9056 (14)	C2A—H2AA	0.9600
Al1—O2^ii^	1.9259 (15)	C2A—H2AB	0.9600
Al1—O2^iii^	1.9259 (15)	C2A—H2AC	0.9600
			
O1—S1—O6	112.13 (9)	O3^ii^—Al2—O4	88.31 (6)
O1—S1—O2	111.68 (10)	O4^ii^—Al2—O4	180.0
O6—S1—O2	110.27 (9)	O3—Al2—O6	88.99 (6)
O1—S1—C1	108.41 (11)	O3^ii^—Al2—O6	91.01 (6)
O6—S1—C1	107.00 (11)	O4^ii^—Al2—O6	89.59 (7)
O2—S1—C1	107.08 (11)	O4—Al2—O6	90.41 (7)
O7A—S2—O4	108.2 (11)	O3—Al2—O6^ii^	91.01 (6)
O7—S2—O4	114.4 (5)	O3^ii^—Al2—O6^ii^	88.99 (6)
O7A—S2—O5	106.5 (9)	O4^ii^—Al2—O6^ii^	90.41 (7)
O7—S2—O5	115.1 (5)	O4—Al2—O6^ii^	89.59 (7)
O4—S2—O5	110.97 (9)	O6—Al2—O6^ii^	180.0
O7A—S2—C2A	112.1 (11)	S1—O2—Al1^iv^	132.00 (10)
O4—S2—C2A	110.1 (11)	Al1—O3—Al2	134.34 (8)
O5—S2—C2A	108.8 (10)	Al1—O3—H3	113.1 (8)
O7—S2—C2	107.9 (5)	Al2—O3—H3	112.3 (8)
O4—S2—C2	103.3 (4)	S2—O4—Al2	138.91 (9)
O5—S2—C2	103.9 (4)	S2—O5—Al1	138.67 (9)
O3—Al1—O3^i^	180.0	S1—O6—Al2	131.92 (9)
O3—Al1—O5	91.67 (6)	S1—C1—H1A	109.5
O3^i^—Al1—O5	88.33 (6)	S1—C1—H1B	109.5
O3—Al1—O5^i^	88.33 (6)	H1A—C1—H1B	109.5
O3^i^—Al1—O5^i^	91.67 (6)	S1—C1—H1C	109.5
O5—Al1—O5^i^	180.0	H1A—C1—H1C	109.5
O3—Al1—O2^ii^	90.97 (6)	H1B—C1—H1C	109.5
O3^i^—Al1—O2^ii^	89.03 (6)	S2—C2—H2A	109.5
O5—Al1—O2^ii^	89.78 (7)	S2—C2—H2B	109.5
O5^i^—Al1—O2^ii^	90.22 (7)	H2A—C2—H2B	109.5
O3—Al1—O2^iii^	89.03 (6)	S2—C2—H2C	109.5
O3^i^—Al1—O2^iii^	90.98 (6)	H2A—C2—H2C	109.5
O5—Al1—O2^iii^	90.22 (7)	H2B—C2—H2C	109.5
O5^i^—Al1—O2^iii^	89.78 (7)	S2—C2A—H2AA	109.5
O2^ii^—Al1—O2^iii^	180.0	S2—C2A—H2AB	109.5
O3—Al2—O3^ii^	180.0	H2AA—C2A—H2AB	109.5
O3—Al2—O4^ii^	88.31 (6)	S2—C2A—H2AC	109.5
O3^ii^—Al2—O4^ii^	91.69 (6)	H2AA—C2A—H2AC	109.5
O3—Al2—O4	91.69 (6)	H2AB—C2A—H2AC	109.5

Symmetry codes: (i) −*x*+1, −*y*, −*z*+1; (ii) −*x*+1, −*y*+1, −*z*+1; (iii) *x*, *y*−1, *z*; (iv) *x*, *y*+1, *z*.

### catena-Poly[aluminium(III)-µ-hydroxido-κ2O:O-di-µ-methanesulfonato-κ4O:O'] Hydrogen-bond geometry (Å, º)

H-bonding distances refer to the major residue.

**Table:**

*D*—H···*A*	*D*—H	H···*A*	*D*···*A*	*D*—H···*A*
C1—H1*B*···O7^v^	0.96	2.62	3.304 (15)	129
C2—H2*B*···O7	0.96	2.72	3.641 (16)	162
O3—H3···O1^vi^	0.94 (1)	1.87 (1)	2.802 (3)	171 (2)

Symmetry codes: (v) −*x*+1, −*y*+1, −*z*+2; (vi) −*x*, −*y*+1, −*z*+1.
